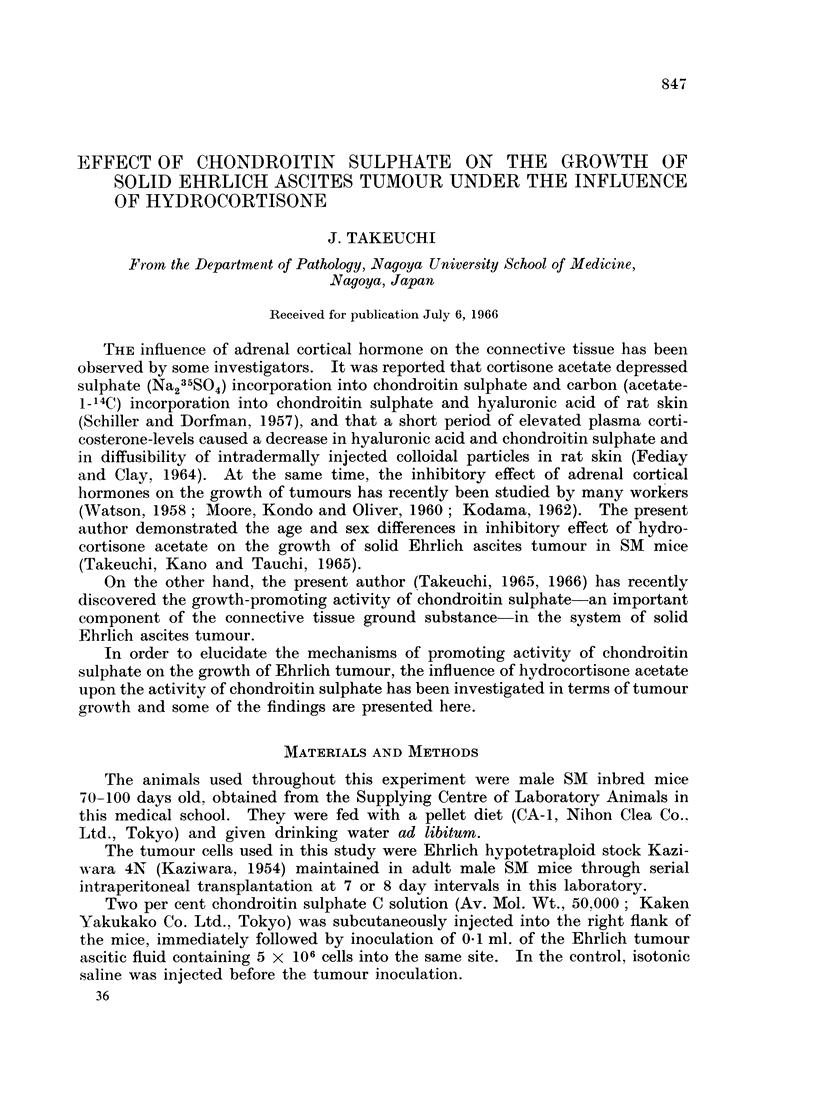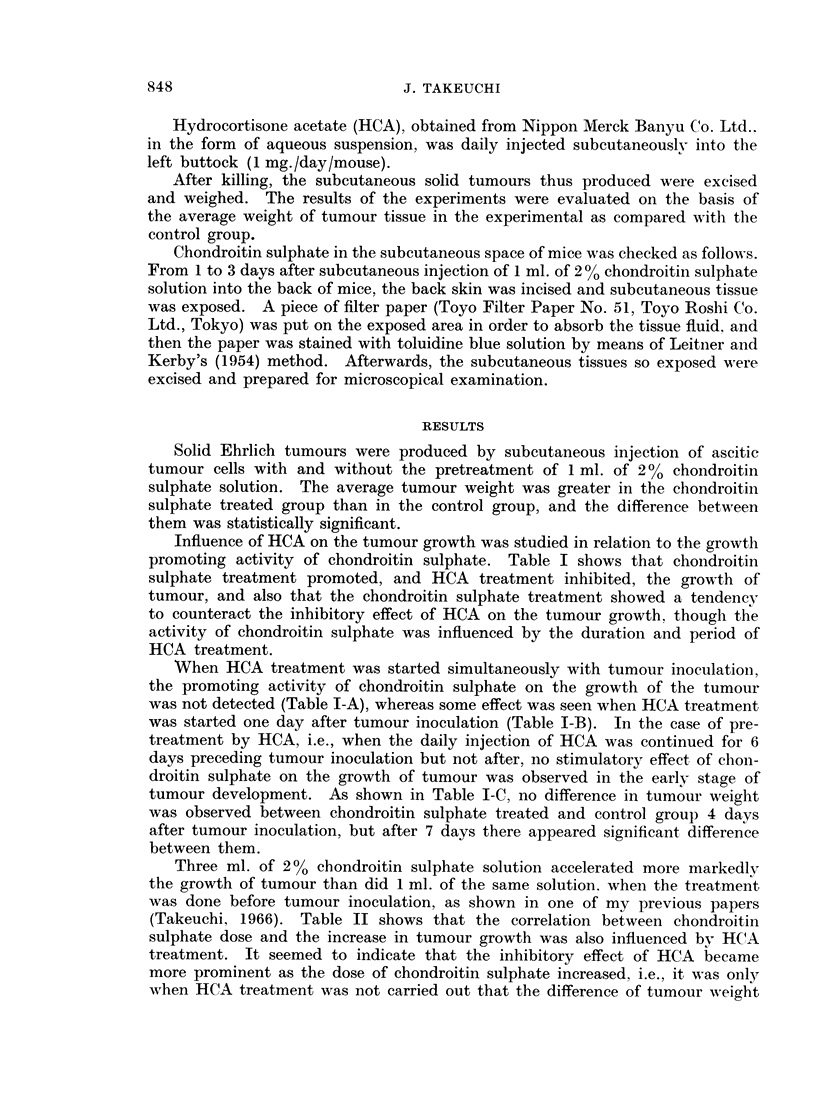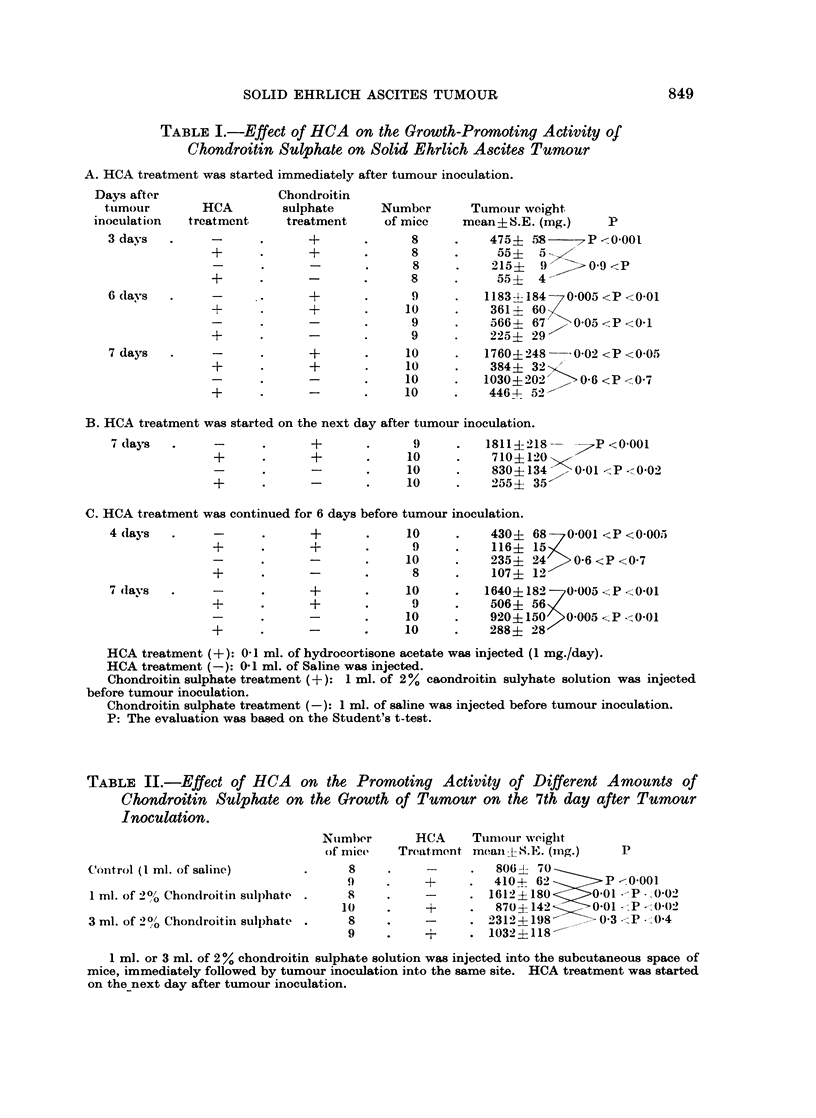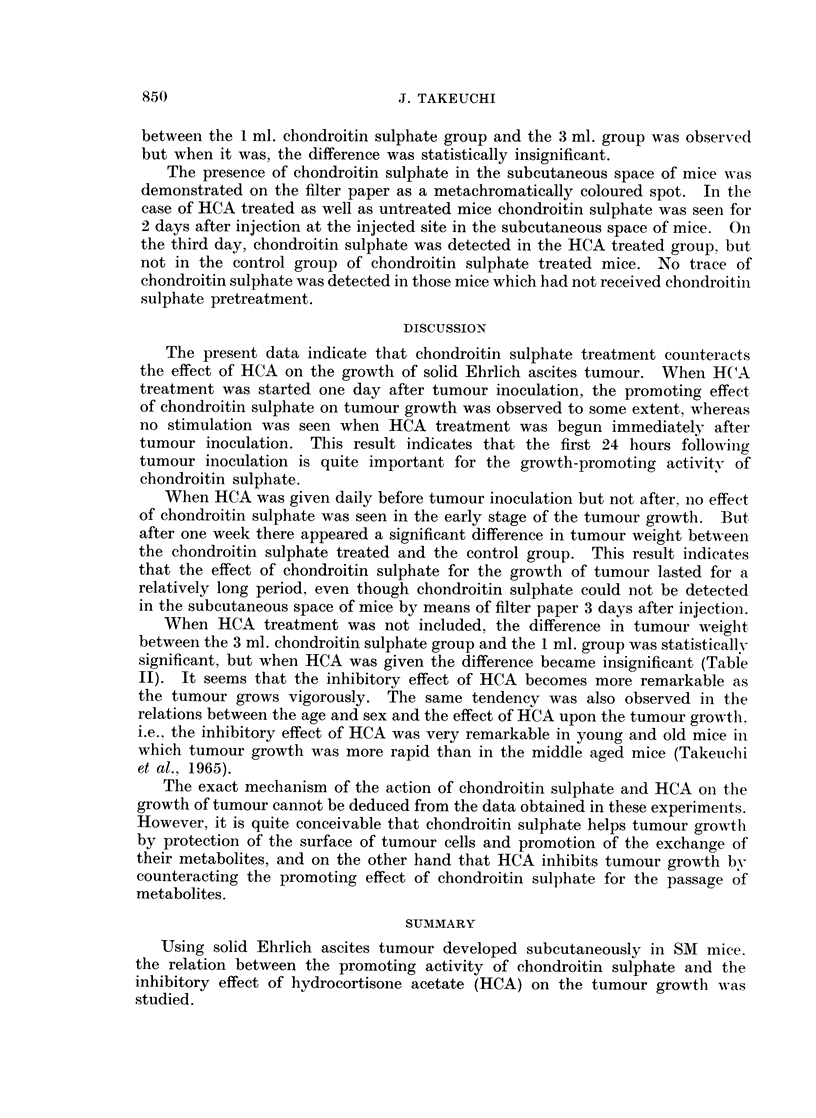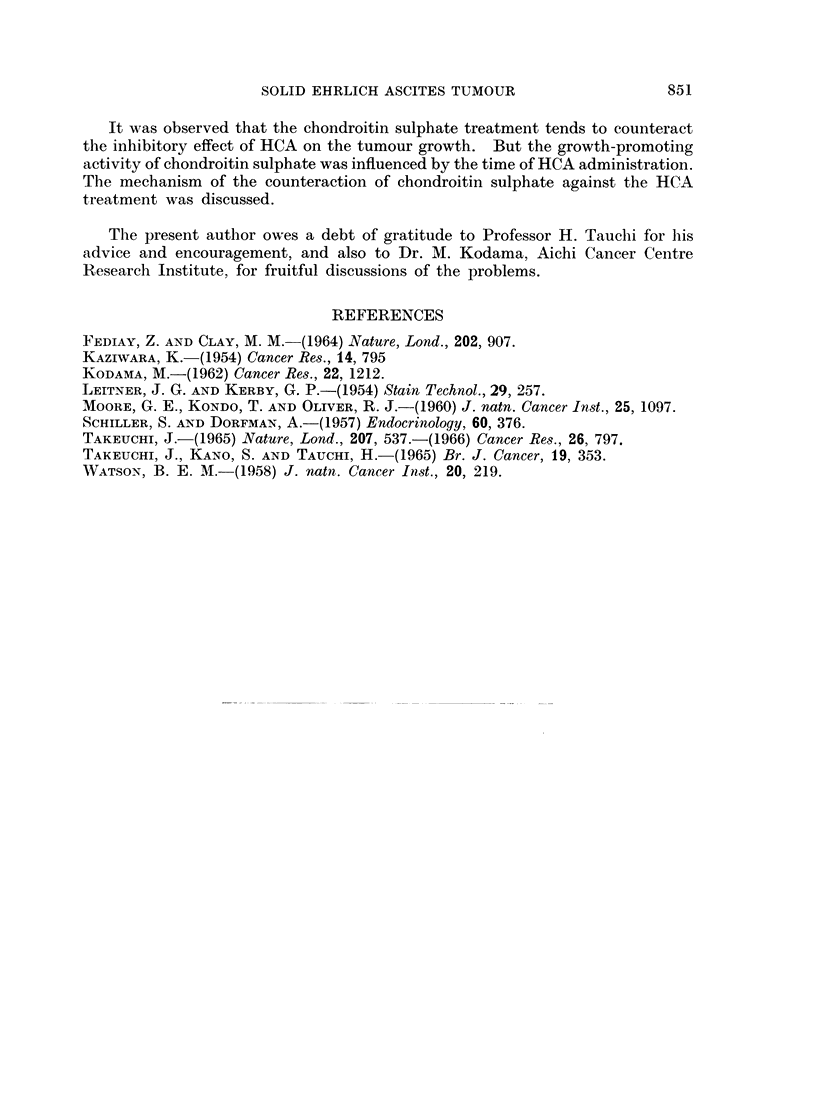# Effect of chondroitin sulphate on the growth of solid Ehrlich ascites tumour under the influence of hydrocortisone.

**DOI:** 10.1038/bjc.1966.97

**Published:** 1966-12

**Authors:** J. Takeuchi


### EFFECT OF CHONDROITIN SULPHATE ON THE GROWTH OF SOLID EHRLICH ASCITES TUMOUR UNDER THE INFLUENCE OF HYDROCORTISONE

J. TAKEUCHI

*From the Department of Pathology, Nagoya University School of Medicine, Nagoya, Japan*

Received for publication July 6, 1966

THE influence of adrenal cortical hormone on the connective tissue has been observed by some investigators. It was reported that cortisone acetate depressed sulphate ($Na_2{}^{35}SO_4$) incorporation into chondroitin sulphate and carbon (acetate-1-$^{14}C$) incorporation into chondroitin sulphate and hyaluronic acid of rat skin (Schiller and Dorfman, 1957), and that a short period of elevated plasma corticosterone-levels caused a decrease in hyaluronic acid and chondroitin sulphate and in diffusibility of intradermally injected colloidal particles in rat skin (Fediay and Clay, 1964). At the same time, the inhibitory effect of adrenal cortical hormones on the growth of tumours has recently been studied by many workers (Watson, 1958 ; Moore, Kondo and Oliver, 1960 ; Kodama, 1962). The present author demonstrated the age and sex differences in inhibitory effect of hydrocortisone acetate on the growth of solid Ehrlich ascites tumour in SM mice (Takeuchi, Kano and Tauchi, 1965).

On the other hand, the present author (Takeuchi, 1965, 1966) has recently discovered the growth-promoting activity of chondroitin sulphate—an important component of the connective tissue ground substance—in the system of solid Ehrlich ascites tumour.

In order to elucidate the mechanisms of promoting activity of chondroitin sulphate on the growth of Ehrlich tumour, the influence of hydrocortisone acetate upon the activity of chondroitin sulphate has been investigated in terms of tumour growth and some of the findings are presented here.

### MATERIALS AND METHODS

The animals used throughout this experiment were male SM inbred mice 70–100 days old, obtained from the Supplying Centre of Laboratory Animals in this medical school. They were fed with a pellet diet (CA-1, Nihon Clea Co., Ltd., Tokyo) and given drinking water *ad libitum.*

The tumour cells used in this study were Ehrlich hypotetraploid stock Kaziwara 4N (Kaziwara, 1954) maintained in adult male SM mice through serial intraperitoneal transplantation at 7 or 8 day intervals in this laboratory.

Two per cent chondroitin sulphate C solution (Av. Mol. Wt., 50,000 ; Kaken Yakukako Co. Ltd., Tokyo) was subcutaneously injected into the right flank of the mice, immediately followed by inoculation of 0·1 ml. of the Ehrlich tumour ascitic fluid containing $5 \times 10^6$ cells into the same site. In the control, isotonic saline was injected before the tumour inoculation.

Hydrocortisone acetate (HCA), obtained from Nippon Merck Banyu Co. Ltd., in the form of aqueous suspension, was daily injected subcutaneously into the left buttock (1 mg./day/mouse).

After killing, the subcutaneous solid tumours thus produced were excised and weighed. The results of the experiments were evaluated on the basis of the average weight of tumour tissue in the experimental as compared with the control group.

Chondroitin sulphate in the subcutaneous space of mice was checked as follows. From 1 to 3 days after subcutaneous injection of 1 ml. of 2% chondroitin sulphate solution into the back of mice, the back skin was incised and subcutaneous tissue was exposed. A piece of filter paper (Toyo Filter Paper No. 51, Toyo Roshi Co. Ltd., Tokyo) was put on the exposed area in order to absorb the tissue fluid, and then the paper was stained with toluidine blue solution by means of Leitner and Kerby's (1954) method. Afterwards, the subcutaneous tissues so exposed were excised and prepared for microscopical examination.

### RESULTS

Solid Ehrlich tumours were produced by subcutaneous injection of ascitic tumour cells with and without the pretreatment of 1 ml. of 2% chondroitin sulphate solution. The average tumour weight was greater in the chondroitin sulphate treated group than in the control group, and the difference between them was statistically significant.

Influence of HCA on the tumour growth was studied in relation to the growth promoting activity of chondroitin sulphate. Table I shows that chondroitin sulphate treatment promoted, and HCA treatment inhibited, the growth of tumour, and also that the chondroitin sulphate treatment showed a tendency to counteract the inhibitory effect of HCA on the tumour growth, though the activity of chondroitin sulphate was influenced by the duration and period of HCA treatment.

When HCA treatment was started simultaneously with tumour inoculation, the promoting activity of chondroitin sulphate on the growth of the tumour was not detected (Table I-A), whereas some effect was seen when HCA treatment was started one day after tumour inoculation (Table I-B). In the case of pre-treatment by HCA, i.e., when the daily injection of HCA was continued for 6 days preceding tumour inoculation but not after, no stimulatory effect of chondroitin sulphate on the growth of tumour was observed in the early stage of tumour development. As shown in Table I-C, no difference in tumour weight was observed between chondroitin sulphate treated and control group 4 days after tumour inoculation, but after 7 days there appeared significant difference between them.

Three ml. of 2% chondroitin sulphate solution accelerated more markedly the growth of tumour than did 1 ml. of the same solution, when the treatment was done before tumour inoculation, as shown in one of my previous papers (Takeuchi, 1966). Table II shows that the correlation between chondroitin sulphate dose and the increase in tumour growth was also influenced by HCA treatment. It seemed to indicate that the inhibitory effect of HCA became more prominent as the dose of chondroitin sulphate increased, i.e., it was only when HCA treatment was not carried out that the difference of tumour weight

TABLE I.—*Effect of HCA on the Growth-Promoting Activity of Chondroitin Sulphate on Solid Ehrlich Ascites Tumour*

A. HCA treatment was started immediately after tumour inoculation.

| Days after tumour inoculation | HCA treatment | Chondroitin sulphate treatment | Number of mice | Tumour weight mean±S.E. (mg.) | P |
|---|---|---|---|---|---|
| 3 days | — | + | 8 | 475± 58 | P <0·001 |
| | + | + | 8 | 55± 5 | |
| | — | — | 8 | 215± 9 | 0·9 <P |
| | + | — | 8 | 55± 4 | |
| 6 days | — | + | 9 | 1183±184 | 0·005 <P <0·01 |
| | + | + | 10 | 361± 60 | |
| | — | — | 9 | 566± 67 | 0·05 <P <0·1 |
| | + | — | 9 | 225± 29 | |
| 7 days | — | + | 10 | 1760±248 | 0·02 <P <0·05 |
| | + | + | 10 | 384± 32 | |
| | — | — | 10 | 1030±202 | 0·6 <P <0·7 |
| | + | — | 10 | 446± 52 | |

B. HCA treatment was started on the next day after tumour inoculation.

| Days after tumour inoculation | HCA treatment | Chondroitin sulphate treatment | Number of mice | Tumour weight mean±S.E. (mg.) | P |
|---|---|---|---|---|---|
| 7 days | — | + | 9 | 1811±218 | P <0·001 |
| | + | + | 10 | 710±120 | |
| | — | — | 10 | 830±134 | 0·01 <P <0·02 |
| | + | — | 10 | 255± 35 | |

C. HCA treatment was continued for 6 days before tumour inoculation.

| Days after tumour inoculation | HCA treatment | Chondroitin sulphate treatment | Number of mice | Tumour weight mean±S.E. (mg.) | P |
|---|---|---|---|---|---|
| 4 days | — | + | 10 | 430± 68 | 0·001 <P <0·005 |
| | + | + | 9 | 116± 15 | |
| | — | — | 10 | 235± 24 | 0·6 <P <0·7 |
| | + | — | 8 | 107± 12 | |
| 7 days | — | + | 10 | 1640±182 | 0·005 <P <0·01 |
| | + | + | 9 | 506± 56 | |
| | — | — | 10 | 920±150 | 0·005 <P <0·01 |
| | + | — | 10 | 288± 28 | |

HCA treatment (+): 0·1 ml. of hydrocortisone acetate was injected (1 mg./day).
HCA treatment (—): 0·1 ml. of Saline was injected.
Chondroitin sulphate treatment (+): 1 ml. of 2% caondroitin sulyhate solution was injected before tumour inoculation.
Chondroitin sulphate treatment (—): 1 ml. of saline was injected before tumour inoculation.
P: The evaluation was based on the Student's t-test.

TABLE II.—*Effect of HCA on the Promoting Activity of Different Amounts of Chondroitin Sulphate on the Growth of Tumour on the 7th day after Tumour Inoculation.*

| | Number of mice | HCA Treatment | Tumour weight mean±S.E. (mg.) | P |
|---|---|---|---|---|
| Control (1 ml. of saline) | 8 | — | 806± 70 | |
| | 9 | + | 410± 62 | P <0·001 |
| 1 ml. of 2% Chondroitin sulphate | 8 | — | 1612±180 | 0·01 <P <0·02 |
| | 10 | + | 870±142 | 0·01 <P <0·02 |
| 3 ml. of 2% Chondroitin sulphate | 8 | — | 2312±198 | 0·3 <P <0·4 |
| | 9 | + | 1032±118 | |

1 ml. or 3 ml. of 2% chondroitin sulphate solution was injected into the subcutaneous space of mice, immediately followed by tumour inoculation into the same site. HCA treatment was started on the next day after tumour inoculation.

between the 1 ml. chondroitin sulphate group and the 3 ml. group was observed but when it was, the difference was statistically insignificant.

The presence of chondroitin sulphate in the subcutaneous space of mice was demonstrated on the filter paper as a metachromatically coloured spot. In the case of HCA treated as well as untreated mice chondroitin sulphate was seen for 2 days after injection at the injected site in the subcutaneous space of mice. On the third day, chondroitin sulphate was detected in the HCA treated group, but not in the control group of chondroitin sulphate treated mice. No trace of chondroitin sulphate was detected in those mice which had not received chondroitin sulphate pretreatment.

### DISCUSSION

The present data indicate that chondroitin sulphate treatment counteracts the effect of HCA on the growth of solid Ehrlich ascites tumour. When HCA treatment was started one day after tumour inoculation, the promoting effect of chondroitin sulphate on tumour growth was observed to some extent, whereas no stimulation was seen when HCA treatment was begun immediately after tumour inoculation. This result indicates that the first 24 hours following tumour inoculation is quite important for the growth-promoting activity of chondroitin sulphate.

When HCA was given daily before tumour inoculation but not after, no effect of chondroitin sulphate was seen in the early stage of the tumour growth. But after one week there appeared a significant difference in tumour weight between the chondroitin sulphate treated and the control group. This result indicates that the effect of chondroitin sulphate for the growth of tumour lasted for a relatively long period, even though chondroitin sulphate could not be detected in the subcutaneous space of mice by means of filter paper 3 days after injection.

When HCA treatment was not included, the difference in tumour weight between the 3 ml. chondroitin sulphate group and the 1 ml. group was statistically significant, but when HCA was given the difference became insignificant (Table II). It seems that the inhibitory effect of HCA becomes more remarkable as the tumour grows vigorously. The same tendency was also observed in the relations between the age and sex and the effect of HCA upon the tumour growth, i.e., the inhibitory effect of HCA was very remarkable in young and old mice in which tumour growth was more rapid than in the middle aged mice (Takeuchi *et al.*, 1965).

The exact mechanism of the action of chondroitin sulphate and HCA on the growth of tumour cannot be deduced from the data obtained in these experiments. However, it is quite conceivable that chondroitin sulphate helps tumour growth by protection of the surface of tumour cells and promotion of the exchange of their metabolites, and on the other hand that HCA inhibits tumour growth by counteracting the promoting effect of chondroitin sulphate for the passage of metabolites.

### SUMMARY

Using solid Ehrlich ascites tumour developed subcutaneously in SM mice. the relation between the promoting activity of chondroitin sulphate and the inhibitory effect of hydrocortisone acetate (HCA) on the tumour growth was studied.

It was observed that the chondroitin sulphate treatment tends to counteract the inhibitory effect of HCA on the tumour growth. But the growth-promoting activity of chondroitin sulphate was influenced by the time of HCA administration. The mechanism of the counteraction of chondroitin sulphate against the HCA treatment was discussed.

The present author owes a debt of gratitude to Professor H. Tauchi for his advice and encouragement, and also to Dr. M. Kodama, Aichi Cancer Centre Research Institute, for fruitful discussions of the problems.

### REFERENCES

FEDIAY, Z. AND CLAY, M. M.—(1964) *Nature, Lond.*, **202**, 907.
KAZIWARA, K.—(1954) *Cancer Res.*, **14**, 795
KODAMA, M.—(1962) *Cancer Res.*, **22**, 1212.
LEITNER, J. G. AND KERBY, G. P.—(1954) *Stain Technol.*, **29**, 257.
MOORE, G. E., KONDO, T. AND OLIVER, R. J.—(1960) *J. natn. Cancer Inst.*, **25**, 1097.
SCHILLER, S. AND DORFMAN, A.—(1957) *Endocrinology*, **60**, 376.
TAKEUCHI, J.—(1965) *Nature, Lond.*, **207**, 537.—(1966) *Cancer Res.*, **26**, 797.
TAKEUCHI, J., KANO, S. AND TAUCHI, H.—(1965) *Br. J. Cancer*, **19**, 353.
WATSON, B. E. M.—(1958) *J. natn. Cancer Inst.*, **20**, 219.